# Adiponectin Expression Is Modulated by Long-Term Physical Activity in Adult Patients Affected by Cystic Fibrosis

**DOI:** 10.1155/2019/2153934

**Published:** 2019-09-09

**Authors:** Rita Polito, Ersilia Nigro, Ausilia Elce, Maria Ludovica Monaco, Paola Iacotucci, Vincenzo Carnovale, Marika Comegna, Monica Gelzo, Federica Zarrilli, Gaetano Corso, Giuseppe Castaldo, Aurora Daniele

**Affiliations:** ^1^Dipartimento di Scienze e Tecnologie Ambientali, Biologiche, Farmaceutiche, Università della Campania “Luigi Vanvitelli”, Caserta 81100, Italy; ^2^CEINGE-Biotecnologie Avanzate, Napoli 80145, Italy; ^3^Dipartimento di Scienze Umanistiche, Università Telematica Pegaso, Napoli 80132, Italy; ^4^Centro Regionale Fibrosi Cistica Adulti, Dipartimento di Scienze Mediche Traslazionali, Università di Napoli Federico II, Napoli 80131, Italy; ^5^Dipartimento di Medicina Molecolare e Biotecnologie Mediche, Università di Napoli Federico II, Napoli 80131, Italy; ^6^Dipartimento di Medicina Clinica e Sperimentale, Università di Foggia, Foggia 71121, Italy

## Abstract

Cystic fibrosis (CF) is a genetic disease characterized by progressive decline of lung function and chronic airway inflammation. Adipose tissue, through adiponectin and leptin, exerts several effects on energy metabolism and inflammatory processes. This study evaluated the levels of adiponectin and leptin in adult healthy subjects, in patients with CF and their correlation with long-term physical activity. CF patients were divided into two groups (sedentary versus active) based on their regular physical activity over 3 years. Anthropometric and serum biochemical profiles of CF patients and controls were evaluated and compared. Total serum adiponectin and leptin levels were measured by ELISA; adiponectin oligomeric profiles were analysed by western blot. Adiponectin levels were significantly higher while leptin levels were lower in patients with CF than in healthy controls. Furthermore, adiponectin was significantly lower in active compared to sedentary CF (*p* = 0.047), while leptin was slightly increased in active compared to sedentary CF. In addition, C-reactive protein levels were significantly lower in active than in sedentary CF patients (*p* = 0.048). Interestingly, only in the active group adiponectin levels were inversely correlated with forced expiratory volume (FEV) 1% decrease/year and FEV1% decrease. Moreover, adiponectin levels negatively correlated with lipid profiles. Our findings indicated that regular, long-term physical activity in CF improves respiratory function, metabolism, and inflammation status. These improvements in patients' conditions are associated with immunometabolic processes involving adiponectin, leptin, and C-reactive protein. Therefore, we propose that both adipokines may be a useful biomarker in the evaluation of metabolic and inflammatory status in patients with CF.

## 1. Introduction

Cystic fibrosis (CF) is the most common autosomal recessive disorder in Caucasian populations. Patients with CF show a severe decline of pulmonary function related to chronic airway inflammation [1]. Recent studies suggest that inflammation occurs very early in pathogenesis and may even occur in the absence of infection; a deregulation of cytokine production and abnormal epithelial host defenses probably underlie and sustain inflammation [2]. A marked and persistent inflammatory process occurs very early in the course of CF and is present in mild as well as in stable disease [3]. Beyond the pulmonary phenotype, patients with CF frequently display alterations in peripheral muscle endurance, likely primarily due to physical inactivity.

The positive effects of physical exercise on body composition, insulin sensitivity, and blood glucose levels have been demonstrated in multiple morbid conditions, including different lung diseases [4, 5]. Respiratory muscle training, physiotherapy, and moderate physical activity have been shown to improve respiratory function in patients with CF [4–6]. Thus, it would be useful to identify biochemical mechanisms involved in such improvement.

Adiponectin is an abundant serum adipokine composing up to 0.05% of total circulating protein [7]. After its secretion by adipocytes, adiponectin oligomerizes in three forms: trimers of low molecular weight (LMW), hexamers of medium molecular weight (MMW), and multimers of high molecular weight (HMW) [8]. HMW multimers are the most active oligomers in their biological properties [8]. In non-obesity-related inflammatory disorders, adiponectin levels are increased [9–11].

Adiponectin plays a role in lung diseases and obesity; indeed, its receptors are expressed in lung epithelium; its serum levels are increased in patients with Chronic Obstructive Pulmonary Disease (COPD) [11]. In addition, data from *in vitro* studies on lung cells were consistent with an anti-inflammatory function of adiponectin [11, 12], and adiponectin-deficient mouse models develop lung function impairment and systemic inflammation [13, 14]. In the development and progression of lung cancer, a pathogenic role of adiponectin was defined by both *in vivo* and *in vitro* studies [15, 16].

Leptin is involved in various inflammatory diseases. In the lung, leptin exerts proinflammatory effects [17]. In the presence of lung inflammatory stimuli, increased leptin concentrations have been associated with symptom intensification and exacerbation [17].

Many studies suggest that exercise is associated with adiponectin and leptin levels in different metabolic diseases [17, 18], but to our knowledge, there are no studies on the effects of exercise on these two adipokines in patients with CF. In this study, we investigated the effects of 3 years of physical training on adiponectin and leptin serum concentrations in a cohort of CF patients compared to a control group of sedentary patients. We also measured correlations between serum adiponectin and leptin levels and biochemical and clinical parameters in the two patient groups. The results were further compared to those from a group of healthy subjects.

## 2. Materials and Methods

### 2.1. Patients

The study population consisted of 116 CF patients who received diagnosis at the regional CF care center of Campania (Southern Italy). All patients who met the diagnostic criteria for CF [19] were over 18 years of age and had pathological SCL (*chloride* > 60 *mEq*/*L*) and two class I-II *CFTR* mutations. Among the 116 patients, 58 regularly performed physical exercise in the last three years (exercise group) and 58 age-matched patients did not perform physical exercise in the last three years. Patients belonging to the exercise group performed physical exercises individually selected by and under the supervision of a team composed of a physician trained in CF, a nutritionist, and a physiotherapist. This team was responsible for monitoring each patient with particular attention to his/her hydration and type of physical activity, to prevent salt depletion syndrome or other complications. Furthermore, the team monitored the compliance of the patients with exercise training. Informed consent was obtained from all participants. Ninety-eight healthy volunteers aged 31.1 ± 7.6 years constituted the control group (*BMI* = 23.4 ± 3.0). The research protocol was conducted in accordance with the principles of the Helsinki II Declaration. According to the current legislation in Italy, we obtained informed consent from each subject for anonymous use of clinical data for research purposes.

### 2.2. Clinical Evaluation

Sweat chloride levels were tested as previously described [20] and a panel of *CFTR* mutations was screened [21]. The best FEV1, expressed as percentage of predicted value for age, according to standardized reference equations for spirometry [19] were recorded. Airway colonization by *Pseudomonas aeruginosa*, pancreatic sufficiency (PS), and liver disease (CFLD) was assessed as previously described [6, 22]. Diagnosis of CFRD was made according to the American Diabetes Association criteria [23].

### 2.3. Anthropometric Measurements

We recorded the height, weight, BMI, waist circumference, and upper arm circumference of CF patients and healthy controls as previously described [6]. Body weight was measured in a fasting state in the morning with a mechanical balance (±0.1 kg, SECA 700, Hamburg DE). BMI was calculated as body weight divided by height squared (kg/m^2^) with categories in accordance with the World Health Organization guidelines [24]. To define subjects with alterations in BMI and circumferences, we used reference intervals normalized to the age of each patient [24].

### 2.4. Biochemical Parameters

Blood samples (*n* = 214) were collected from the participants after an overnight fast, and serum samples were separated. Serum albumin, insulin, C-reactive protein, glucose, total cholesterol, HDL and LDL cholesterol, and triglycerides were measured as previously described [6]; all assays were performed with an automated biochemistry analyser (Architect *ci*16200 Integrated System, Abbott Diagnostics, Rome, Italy). The HOMA index was calculated as *fasting* *glucose* × *fasting* *insulin*/22.5 [23]. Concentrations of total adiponectin in serum were measured in triplicate by an enzyme-linked immunosorbent assay (ELISA) as previously described [25]. A Sandwich-ELISA assay was used to measure leptin concentration in serum samples (Elabscience, Houston, Texas, USA).

### 2.5. Western Blotting Analysis

Serum samples from all participants were quantified for total proteins by Bradford assay (Bio-Rad, Hercules, CA, USA), and 10 *μ*g of total protein was heated in 1X Laemmli buffer at 95°C for 10 min and loaded on 10% SDS-PAGE gels as previously described [11]. The immunoblots were developed by ECL (Amersham Biosciences, Piscataway, NJ, USA) with the use of Kodak BioMax Light film, digitalised with a scanner (1200 dpi), and analysed by densitometry with ImageJ software (https://imagej.nih.gov/ij/). Each sample was tested three times in duplicate.

### 2.6. Statistical Analysis

All data are expressed as *means* ± *SD*. The Kolmogorov-Smirnov test was used to test the normality of distributions. Student's *t*-test or the Mann-Whitney *U* test for independent samples were used to compare parametric and nonparametric numerical data, respectively. Frequencies of categorical variables were compared by Chi-square tests. Adiponectin serum concentrations were correlated by Pearson's or Spearman's rho tests, according to data distribution. A *p* value < 0.05 was considered statistically significant. Multiple comparisons on western blotting experiments were performed by ANOVA tests. All statistical analyses were performed with the Statistical Package for Social Sciences software for Windows (SPSS Inc.).

## 3. Results

### 3.1. Anthropometric and Biochemical Features of Patients with CF

Anthropometric and serum biochemical profiles of CF patients and control healthy subjects are reported in Table 1.

All subjects were matched for age and sex. Data are shown in Table 1. BMI values were significantly lower in patients with CF compared to controls. CF patients had significantly lower cholesterol lipid and significantly higher glucose levels than controls. Adiponectin serum levels were increased in CF patients *vs.* controls, while leptin levels are significantly decreased in CF patients *vs.* controls.

After the comparison to healthy subjects, we divided the CF patients into two age- and sex-matched subgroups and compared anthropometrical, biochemical, and disease-specific parameters between patients that regularly performed physical exercise in the last three years and sedentary patients.  Table 2 shows that these two subgroups (active and sedentary) significantly differed for both FEV1% decrease/year and FEV1% decrease. In the active group, triglyceride levels were significantly lower than in the sedentary group. Serum adiponectin levels were significantly lower in active versus sedentary CF patients, respectively. On the contrary, leptin levels are slightly decreased in sedentary CF patients *vs.* active patients, although the difference is not significant. Finally, C-reactive protein levels were significantly lower in the active group than in the sedentary group.

### 3.2. Adiponectin Correlations to Biochemical and Clinical Parameters in CF Patients

Table 3 shows the correlations observed between adiponectin, biochemical, and FEV parameters in active and sedentary CF patients. Adiponectin correlated negatively with both FEV1% decrease per year as well as with FEV% decrease only in the active group. In addition, adiponectin correlated negatively with lipid biomarkers; in particular, we observed a negative correlation with total cholesterol (chol), LDL, and non-HDL chol in both groups. Finally, serum triglyceride levels correlated negatively with adiponectin levels only in the active group.

### 3.3. Adiponectin Oligomerization State Analysis in Serum by Western Blotting

**Figure 1:**
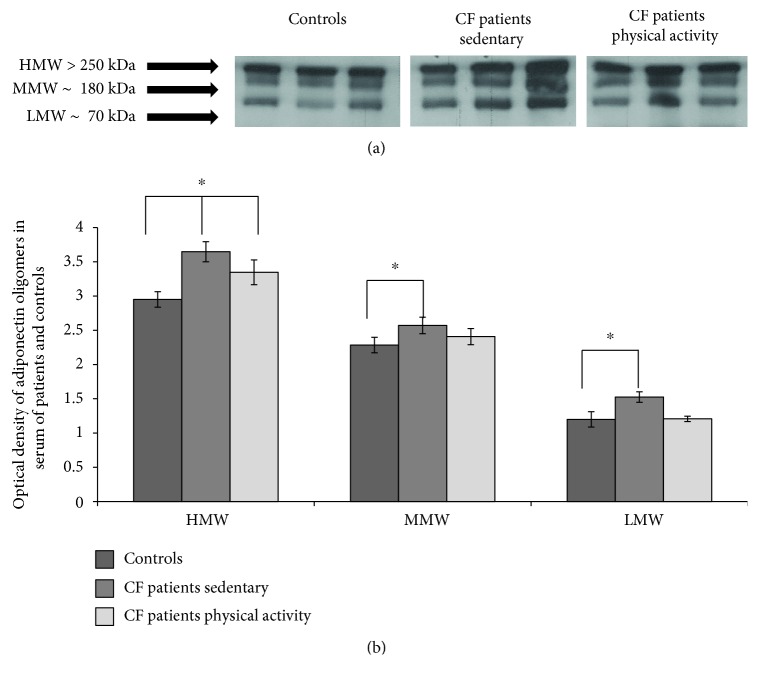
Adiponectin HMW oligomer levels are decreased by long-term physical activity. (a) Representative images from blots of serum adiponectin oligomers (HMW, MMW, and LMW) from healthy controls, CF sedentary patients, and CF active patients; (b) quantification of adiponectin oligomers in serum samples. Each sample was tested three times in duplicate, and values are reported as percentage compared to controls. Multiple comparisons were performed by ANOVA.

The distribution of serum adiponectin in sedentary and active CF patients was analysed and compared to the healthy control group by western blot (Figure 1). Three bands corresponding to HMW (≥250 kDa), MMW (180 kDa), and LMW (70 kDa) oligomers were evident in serum from both controls and patients (Figure 1(a)). The densitometric evaluation of adiponectin oligomers in serum showed an increased expression of the HMW, MMW, and LMW oligomers in CF patients compared to controls (Figure 1(b)) (*p* < 0.05). On the other hand, adiponectin oligomers were only slightly more highly expressed (not statistically significant) in active CF patients compared to healthy controls but significantly lower expression compared to sedentary CF patients.

## 4. Discussion

Serum adiponectin levels were significantly higher while leptin levels were significantly decreased in CF patients compared to healthy controls. Furthermore, patients with CF that performed 3 years of regular physical activity had adiponectin concentrations significantly lower than those of sedentary patients; in parallel, serum C-reactive protein levels were significantly lower in the active CF group than in the sedentary CF group. Interestingly, in the active CF group, adiponectin levels were negatively correlated with FEV1% decrease per year, FEV1% decrease, and serum CRP. In addition, adiponectin levels were negatively correlated with lipid profiles in the entire cohort of CF patients. Regarding leptin levels, patients with CF that performed 3 years of regular physical activity had increased levels compared to sedentary patients, although data are not significant.

CF is characterized by systemic inflammation associated with progressive airflow obstruction (FEV1), airway neutrophilic inflammation, and recurrent exacerbations; these phenotypical and pathologic features are shared with COPD [10]. In this context, looking at adiponectin and leptin as important modulators of inflammatory processes implicated in airway pathophysiology, we found that serum levels of adiponectin were significantly increased in CF patients compared to healthy controls while leptin levels were significantly decreased in the total cohort of CF patients compared to healthy controls. Moriconi et al. reported increased serum adiponectin in CF patients than in controls, independently of other confounders [26]. In line with these results, significantly increased adiponectin concentrations and a direct correlation to the severity of disease have been observed in patients affected by COPD [11, 27–30]. In addition, a relationship between adiponectin concentration and lung function decline has been reported [31]. More recently, an involvement of adiponectin has been described during the pathogenesis of emphysema [16, 28]. Contrasting results are observed for leptin levels in patients with CF [32–36].

Previously, we reported that long-term physical exercise had significantly beneficial effects on FEV1% decline, BMI, waist and arm circumferences, and lipid and glucose metabolism in CF patients [6]. In the present study, we examined the effects of physical activity on adiponectin and leptin levels in such patients. Interestingly, we observed that the adiponectin levels significantly decreased with 3 years of regular exercise while leptin levels are increased, although not significant. In particular, in patients with CF, long-term physical exercise produces both an amelioration of the pulmonary phenotype and an improvement of systemic inflammation as demonstrated by the FEV1% decrease, by CRP and adiponectin reduction, and the increase of leptin serum levels. Furthermore, the negative correlation between adiponectin and FEV1% decrease/year and FEV1% decrease observed in the active group of CF patients is consistent with this hypothesis. On the other hand, adiponectin levels are usually elevated in classic chronic inflammatory/immune diseases such as multiple sclerosis, common variable immunodeficiency, and inflammatory bowel disease [9, 37–39]. In this context, our data highlight the key role of adiponectin in lung pathologies as a crosstalk between lung and adipose tissue. Our data also underline a correlation between adiponectin and the lipid profile of CF patients. In our cohort of CF patients performing regular exercise for 3 years, physical training induces the mobilization of triglycerides, improving the metabolic profile that correlates with adiponectin levels. Panagopoulou et al. previously demonstrated that adiponectin is higher in CF patients than in controls with lower levels in malnourished young patients and higher in patients with normal nutrition. The authors concluded that this may be attributed to the energy deficit due to the disease [40]. Ziai et al. demonstrated that the association between total adiponectin and glucose and insulin metabolism is different in CF patients compared to controls, although they found similar levels of adiponectin between CF patients and controls [33].

In literature, conflicting data are present on leptin concentrations and its role in CF patients [32–36]. To our knowledge, no data about physical activity on leptin levels are reported. In this study, we observed that leptin levels are slightly increased in active CF vs. sedentary patients. These results are in line with previously reported data that observed different concentrations of leptin according to CF disease phenotype and concluded that severe CF patients had significantly decreased leptin compared to controls [35]. This data indicated that leptin may be a further indicator of inflammatory status in patients with CF. However, a limitation of the study is represented by the limited number of CF patients and controls analysed.

## 5. Conclusion

In conclusion, our results indicate that in CF patients, long-term physical exercise ameliorates the pulmonary phenotype and improves the metabolic profile and systemic inflammation as shown by serum CRP reduction, adiponectin decrease, and leptin increase. Recently, immunometabolic pathomechanisms have been identified as important factors determining and modulating lung function and disease [41]. Therefore, both adipokines appear to be an attractive biomarker for monitoring CF progression. Further studies are needed to define the molecular mechanisms through which adiponectin and leptin modulates such effects.

## Figures and Tables

**Table 1:** Sex distribution, anthropometric data, and serum biochemical parameters in patients with CF and in control subjects, CN (mean values and standard deviation).

	CN subjects (*n* = 98)	CF patients (*n* = 116)	*p* value
Age (years)	31.1 (7.6)	30.9 (8.7)	*n.s.* ^∗^
Sex male/female	55/43	59/57	*n.s.* ^∗∗^
Body mass index (kg/m^2^)	23.4 (3.0)	22.4 (3.6)	*.001* ^∗^
Total chol (mg/dL)	190.2 (35.3)	140.9 (36.6)	*.000*
Triglycerides (mg/dL)	86.5 (53.7)	79.6 (29.5)	*n.s.* ^∗^
HDL chol (mg/dL)	56.6 (15.2)	47.0 (14.3)	*.000*
LDL chol (mg/dL)	114.8 (30.2)	78.0 (28.0)	*.000*
Non-HDL chol (mg/dL)	133.6 (35.9)	93.9 (31.2)	*.000*
Tot/HDL chol ratio (%)	3.6 (1.2)	3.1 (0.8)	*.010* ^∗^
Non-HDL/HDL chol ratio (%)	2.6 (1.2)	2.1 (0.8)	*.010* ^∗^
Serum fasting glucose (mg/dL)	83.2 (12.8)	90.8 (17.6)	*.000* ^∗^
Albumin (g/dL)	4.7 (0.4)	4.3 (0.4)	*.000*
Adiponectin (*μ*g/mL)	10.2 (1.6)	13.2 (2.2)	*.000*
Leptin (ng/mL)	12.0 (6.1)	9.2 (11.2)	*.003* ^∗^

*p* values by *t*-test, ^∗^Mann-Whitney *U* test or ^∗∗^Chi-square test; *n.s*.: not significant.

**Table 2:** Sex distribution, anthropometric data, biochemical parameters, and FEV values in patients with CF divided on the basis of physical activity (mean values and standard deviation).

	CF physical activity group (*n* = 58)	CF sedentary group (*n* = 58)	*p* value
Age (years)	30.7 (8.3)	31.1 (9.2)	*n.s.*
Sex (male/female)	29/29	30/28	*n.s.* ^∗∗^
Body mass index (kg/m^2^)	22.4 (3.2)	22.4 (4.0)	*n.s.* ^∗^
Waist circumference (cm)	80.0 (8.9)	81.5 (9.7)	*n.s.*
Hip circumference (cm)	93.4 (7.6)	92.8 (9.1)	*n.s.*
Waist/hip ratio (%)	86.2 (8.9)	88.1 (8.4)	*n.s.*
FEV1% decrease/year	0.46 (3.58)	1.13 (5.01)	*.044*
FEV1% decrease (%)	0.87 (4.77)	1.03 (8.02)	*.008*
Total cholesterol	140.3 (37.3)	141.6 (36.2)	*n.s.*
Triglycerides (mg/dL)	72.7 (20.6)	86.6 (35.2)	*.012*
Serum fasting glucose (mg/dL)	90.0 (21.4)	91.5 (13.1)	*n.s.* ^∗^
Insulinemia (mg/dL)	8.1 (7.2)	10.5 (9.2)	*n.s.* ^∗^
HOMA-IR	1.8 (1.5)	2.4 (2.1)	*n.s.* ^∗^
FGIR	17.5 (10.3)	14.6 (10.3)	*n.s.*
Adiponectin (*μ*g/mL)	12.8 (2.1)	13.7 (2.2)	*.047*
Leptin (ng/mL)	10.8 (13.7)	7.7 (8.1)	*n.s.* ^∗^
C-reactive protein (mg/L)	3.65 (3.98)	6.33 (8.52)	*.048*

*p* values by *t*-test, ^∗^Mann-Whitney *U* test or ^∗∗^Chi-square test; n.s.: not significant.

**Table 3:** The statistically serum adiponectin correlations in CF patients, divided according to physical activity.

	CF physical activity group (*n* = 50)	CF sedentary group (*n* = 49)
FEV1% decrease/year	*.013 (-)*	
FEV1% decrease (%)	*.025 (-)*	
Total cholesterol	*.024 (-)*	*.022 (-)*
Triglycerides	*.003 (-)*	
LDL cholesterol	.*023 (-)*	*.024 (-)*
Non-HDL cholesterol	*.013 (-)*	*.043 (-)*
C-reactive protein	*.011 (+)* ^∗^	

*p* values by Pearson correlation or ^∗^by Spearman's rho correlation.

## Data Availability

No sensitive data concerning the patients has been included in the article.

## References

[1] Aghasafari P., George U., Pidaparti R. (2019). A review of inflammatory mechanism in airway diseases. *Inflammation Research*.

[2] Palaniyar N., Mall M. A., Taube C., Worgall S., Grasemann H. (2015). New developments in cystic fibrosis airway inflammation. *Mediators of Inflammation*.

[3] Cohen-Cymberknoh M., Kerem E., Ferkol T., Elizur A. (2013). Airway inflammation in cystic fibrosis: molecular mechanisms and clinical implications. *Thorax*.

[4] Zhao R. R., O’Sullivan A. J., Fiatarone Singh M. A. (2018). Exercise or physical activity and cognitive function in adults with type 2 diabetes, insulin resistance or impaired glucose tolerance: a systematic review. *European Review of Aging and Physical Activity*.

[5] Spruit M. A., Pitta F., McAuley E., ZuWallack R. L., Nici L. (2015). Pulmonary rehabilitation and physical activity in patients with chronic obstructive pulmonary disease. *American Journal of Respiratory and Critical Care Medicine*.

[6] Elce A., Nigro E., Gelzo M. (2018). Supervised physical exercise improves clinical, anthropometric and biochemical parameters in adult cystic fibrosis patients: a 2-year evaluation. *The Clinical Respiratory Journal*.

[7] Liu M., Liu F. (2010). Transcriptional and post-translational regulation of adiponectin. *Biochemical Journal*.

[8] Nigro E., Scudiero O., Monaco M. L. (2014). New insight into adiponectin role in obesity and obesity-related diseases. *BioMed Research International*.

[9] Daniele A., de Rosa A., de Cristofaro M. (2011). Decreased concentration of adiponectin together with a selective reduction of its high molecular weight oligomers is involved in metabolic complications of myotonic dystrophy type 1. *European Journal of Endocrinology*.

[10] Signoriello E., Lus G., Polito R. (2019). Adiponectin profile at baseline is correlated to progression and severity of multiple sclerosis. *European Journal of Neurology*.

[11] Daniele A., de Rosa A., Nigro E. (2012). Adiponectin oligomerization state and adiponectin receptors airway expression in chronic obstructive pulmonary disease. *The International Journal of Biochemistry & Cell Biology*.

[12] Nigro E., Scudiero O., Sarnataro D. (2013). Adiponectin affects lung epithelial A549 cell viability counteracting TNF*α* and IL-1*β* toxicity through AdipoR1. *The International Journal of Biochemistry & Cell Biology*.

[13] Summer R., Little F. F., Ouchi N. (2008). Alveolar macrophage activation and an emphysema-like phenotype in adiponectin-deficient mice. *American Journal of Physiology. Lung Cellular and Molecular Physiology*.

[14] Summer R., Fiack C. A., Ikeda Y. (2009). Adiponectin deficiency: a model of pulmonary hypertension associated with pulmonary vascular disease. *American Journal of Physiology. Lung Cellular and Molecular Physiology*.

[15] Illiano M., Nigro E., Sapio L. (2017). Adiponectin down-regulates CREB and inhibits proliferation of A549 lung cancer cells. *Pulmonary Pharmacology & Therapeutics*.

[16] Boura P., Loukides S., Grapsa D., Achimastos A., Syrigos K. (2015). The diverse roles of adiponectin in non-small-cell lung cancer: current data and future perspectives. *Future Oncology*.

[17] Yu N., Ruan Y., Gao X., Sun J. (2017). Systematic review and meta-analysis of randomized, controlled trials on the effect of exercise on serum leptin and adiponectin in overweight and obese individuals. *Hormone and Metabolic Research*.

[18] Farrell P. M., Rosenstein B. J., White T. B. (2008). Guidelines for diagnosis of cystic fibrosis in newborns through older adults: Cystic Fibrosis Foundation consensus report. *The Journal of Pediatrics*.

[19] VA L. G., Yankaskas J. R., Quittell L. M., Marshall B. C., Mogayzel P. J., Cystic Fibrosis Foundation (2007). Diagnostic sweat testing: the Cystic Fibrosis Foundation guidelines. *The Journal of Pediatrics*.

[20] Tomaiuolo R., Sangiuolo F., Bombieri C. (2008). Epidemiology and a novel procedure for large scale analysis of CFTR rearrangements in classic and atypical CF patients: a multicentric Italian study. *Journal of Cystic Fibrosis*.

[21] Quanjer P. H., Stanojevic S., Cole T. J. (2012). Multi-ethnic reference values for spirometry for the 3–95-yr age range: the global lung function 2012 equations. *The European Respiratory Journal*.

[22] Debray D., Kelly D., Houwen R., Strandvik B., Colombo C. (2011). Best practice guidance for the diagnosis and management of cystic fibrosis-associated liver disease. *Journal of Cystic Fibrosis*.

[23] Ascaso J. F., Pardo S., Real J. T., Lorente R. I., Priego A., Carmena R. (2003). Diagnosing insulin resistance by simple quantitative methods in subjects with normal glucose metabolism. *Diabetes Care*.

[24] http://www.euro.who.int/en/health-topics/disease-prevention/nutrition/a-healthy-lifestyle/body-mass-index-bmi

[25] Nigro E., Piombino P., Scudiero O. (2015). Evaluation of salivary adiponectin profile in obese patients. *Peptides*.

[26] Moriconi N., Kraenzlin M., Müller B. (2006). Body composition and adiponectin serum concentrations in adult patients with cystic fibrosis. *The Journal of Clinical Endocrinology and Metabolism*.

[27] Jaswal S., Saini V., Kaur J., Gupta S., Kaur H., Garg K. (2018). Association of adiponectin with lung function impairment and disease severity in chronic obstructive pulmonary disease. *International Journal of Applied & Basic Medical Research*.

[28] Kanazawa H., Yoshikawa T. (2012). Association of plasma adiponectin levels with cellular hydration state measured using bioelectrical impedance analysis in patients with COPD. *International Journal of Chronic Obstructive Pulmonary Disease*.

[29] Shaw J. G., Vaughan A., Dent A. G. (2014). Biomarkers of progression of chronic obstructive pulmonary disease (COPD). *Journal of Thoracic Disease*.

[30] Carolan B. J., Kim Y. I., Williams A. A. (2013). The association of adiponectin with computed tomography phenotypes in chronic obstructive pulmonary disease. *American Journal of Respiratory and Critical Care Medicine*.

[31] Sato K., Shibata Y., Abe S. (2014). Association between plasma adiponectin levels and decline in forced expiratory volume in 1 s in a general Japanese population: the Takahata study. *International Journal of Medical Sciences*.

[32] Monajemzadeh M., Ashtiani M. T. H., Sadrian E. (2013). Variation in plasma leptin levels in young Iranian children with cystic fibrosis. *Archives of Medical Science*.

[33] Ziai S., Belson L., Malet A. (2012). The association between leptin and insulin levels in adults with cystic fibrosis. *Diabetes & Metabolism*.

[34] Cohen R. I., Tsang D., Koenig S., Wilson D., McCloskey T., Chandra S. (2008). Plasma ghrelin and leptin in adult cystic fibrosis patients. *Journal of Cystic Fibrosis*.

[35] Boguszewski M. C. S., Kamoi T. O., Bento Radominski R. (2007). Insulin-like growth factor-1, leptin, body composition, and clinical status interactions in children with cystic fibrosis. *Hormone Research*.

[36] Schmitt-Grohé S., Hippe V., Igel M. (2006). Serum leptin and cytokines in whole blood in relation to clinical and nutritional status in cystic fibrosis. *Journal of Pediatric Gastroenterology and Nutrition*.

[37] Pecoraro A., Nigro E., Polito R. (2017). Total and high molecular weight adiponectin expression is decreased in patients with common variable immunodeficiency: correlation with Ig replacement therapy. *Frontiers in Immunology*.

[38] Morshedzadeh N., Rahimlou M., Asadzadeh Aghdaei H., Shahrokh S., Reza Zali M., Mirmiran P. (2017). Association between adipokines levels with inflammatory bowel disease (IBD): systematic reviews. *Digestive Diseases and Sciences*.

[39] Takeda Y., Nakanishi K., Tachibana I., Kumanogoh A. (2012). Adiponectin: a novel link between adipocytes and COPD. *Vitamins and Hormones*.

[40] Panagopoulou P., Fotoulaki M., Manolitsas A., Pavlitou-Tsiontsi E., Tsitouridis I., Nousia-Arvanitakis S. (2008). Adiponectin and body composition in cystic fibrosis. *Journal of Cystic Fibrosis*.

[41] Bruzzaniti S., Bocchino M., Santopaolo M. (2019). An immunometabolic pathomechanism for chronic obstructive pulmonary disease. *Proceedings of the National Academy of Sciences*.

